# Foodborne antibiotics enrich human gut microbiota with pathogens producing extended-spectrum β-lactamases and carbapenemases

**DOI:** 10.1093/ismejo/wrag008

**Published:** 2026-01-26

**Authors:** Daniel Martak, Thibault Bourdin, Benoit Valot, Audrey Laboissière, Frédéric Lirussi, Xavier Bertrand, Edward Topp, Didier Hocquet

**Affiliations:** Université Marie et Louis Pasteur, CNRS, Chrono-environnement (UMR 6249), Besançon F-25000, France; Hygiène Hospitalière, Centre Hospitalier Universitaire, F-25030 Besançon, France; Université Marie et Louis Pasteur, CNRS, Chrono-environnement (UMR 6249), Besançon F-25000, France; Hygiène Hospitalière, Centre Hospitalier Universitaire, F-25030 Besançon, France; Université Marie et Louis Pasteur, CNRS, Chrono-environnement (UMR 6249), Besançon F-25000, France; Université Marie et Louis Pasteur, CNRS, Chrono-environnement (UMR 6249), Besançon F-25000, France; Plateforme PACE, Laboratoire de Pharmacologie-Toxicologie, Centre Hospitalier Universitaire, Besançon F-25000, France; Université Marie et Louis Pasteur, CNRS, Chrono-environnement (UMR 6249), Besançon F-25000, France; Hygiène Hospitalière, Centre Hospitalier Universitaire, F-25030 Besançon, France; Agroécologie UMR1347, INRAE Centre Bourgogne-Franche-Comté, Université de Bourgogne, Dijon F-21000, France; Department of Biology, University of Western Ontario, London, ON N6A 5B7, Canada; Université Marie et Louis Pasteur, CNRS, Chrono-environnement (UMR 6249), Besançon F-25000, France; Hygiène Hospitalière, Centre Hospitalier Universitaire, F-25030 Besançon, France; Centre de Ressources Biologiques - Filière Microbiologique, Centre Hospitalier Universitaire, Besançon F-25030, France

**Keywords:** antibiotics, antimicrobial resistance, Gram-negative bacilli, epidemiology, food, gut microbiota

## Abstract

Antimicrobial resistance is a serious global health threat, yet the drivers of its spread among humans are not fully understood. Antibiotics can enter the human gastrointestinal tract through the food chain, leading to the presence of low concentrations in the gut microbiota. However, the role of such traces in promoting the implantation of drug-resistant pathogens in the gut microbiota has never been explored in a controlled experimental setting. Using an *in vitro* model of the human gut microbiota, we tested whether traces of 19 antibiotics used in both human and veterinary medicine, alone or in combination, lead to the enrichment of Gram-negative pathogens producing extended-spectrum β-lactamases or carbapenemases. Twenty-eight strains of Gram-negative pathogens epidemic in humans (10 *Escherichia coli*, 6 *Klebsiella pneumoniae*, 5 *Enterobacter hormaechei*, 4 *Acinetobacter baumannii*, 3 *Pseudomonas aeruginosa*) were tested. We found that antibiotics at levels similar to those measured in the feces of healthy individuals (fluoroquinolones, 1–100 μg L^−1^; trimethoprim, 100 μg L^−1^; a mixture of fifteen veterinary antibiotics, 10–20 μg L^−1^) enriched the human gut microbiota with those resistant pathogens. Overall, the present study indicates that dietary consumption of some antibiotics can result in concentrations in the human colon sufficiently high to favor the implantation of exogenous antibiotic-resistant pathogens. These findings highlight the need to reassess permissible antibiotic concentrations in food and critically evaluate agricultural practices contributing to the contamination of animal- and plant-based products.

## Graphical Abstract

**Graphical Abstract:**
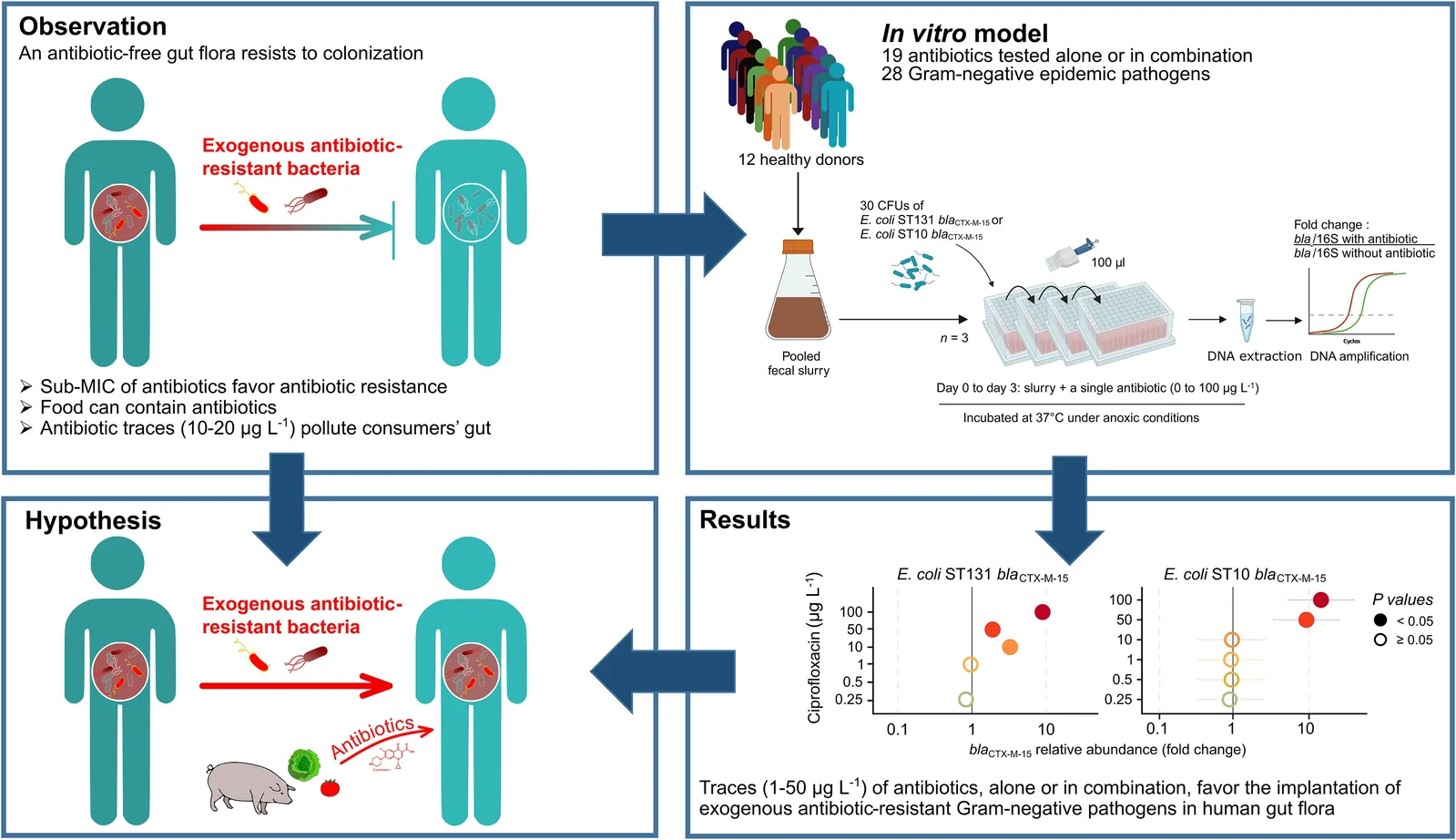

## Introduction

In 2019, the World Health Organization (WHO) declared antimicrobial resistance (AMR) one of the top ten global public health threats facing humanity [1]. AMR is steadily rising globally, especially in low and middle-income countries [2]. Among the drug-resistant pathogens, Gram-negative bacilli resistant to third-generation cephalosporins and/or to carbapenems are of major public health importance, together responsible for more than 300 000 deaths globally in 2019 [3].

Intestinal colonization by these β-lactamase–producing bacteria represents the first step in their pathogenesis and is normally impeded by a healthy gut microbiota [4, 5]. In contrast, antibiotic-resistant pathogens can readily colonize the gastrointestinal tract in animal models following microbiota disruption induced by a single antibiotic dose [6]. In humans, the response to exposure to a given antibiotic can vary between individuals, likely due to multiple factors including the inherent resistance and resilience of the gut microbiota, a history of previous antibiotic use, and co-morbidities [7, 8]. Overall, an understanding of the specific impacts of antibiotic use on the gut microbiota and the consequent likelihood of an individual becoming vulnerable to colonization with an antibiotic-resistant pathogen needs to be considered within this complexity.

One factor that will obviously influence the impact of an antibiotic on the gut microbiota is the dose, and the concentration that the drug consequently reaches in the gastrointestinal tract over a specified period of time. In general, observations on humans and experiments with animals concern clinical antibiotic doses intended to reach or exceed the minimum inhibitory concentration (MIC) in the tissue or organ infected [9]. Yet, humans are exposed to low doses of antibiotics through other pathways, through the consumption of contaminated water or, no doubt more commonly, animal- or plant-based foods containing antibiotic residues [10–12]. Antibiotics are widely used in food-animal production systems [13] and fertilization of crop ground with animal or human fecal waste can contaminate the harvested products [14]. β-lactamase-producing pathogens are very frequently co-resistant to other antibiotic compounds that can contaminate food products (e.g. fluoroquinolones, sulfonamides, trimethoprim) [15]. This co-resistance suggests that exposure to any of these antibiotics could select for ESBL- and carbapenemase-producing bacteria.

Although inter-individual transmission is the major source of human contamination with drug-resistant Gram-negative bacilli [16, 17], the factors that drive the spread of AMR in humans are far from being fully understood. Yet, a deep understanding of the mechanisms and drivers of AMR transmission is needed to implement innovative and effective strategies [18–20]. Although antibiotics can enter the human gastrointestinal tract via the food chain [21–23], the role of food-borne antibiotic residues in the implantation of antibiotic-resistant pathogens in the human gut has never been considered. Here, we hypothesized that concentrations of antibiotics in the range of those measured in the feces of healthy people exposed to antibiotic-containing food, can favor the implantation and development of antibiotic-resistant pathogens in the gut microbiota. Using an *in vitro* model of the human gut microbiota, we tested the ability of low concentrations of 19 clinical and veterinary antibiotics, alone or in combination, to enrich 28 widespread clones of Gram-negative pathogens carrying genes encoding extended-spectrum ꞵ-lactamases (*bla*_ESBL_) and carbapenemases (*bla*_CARBA_).

## Material and methods

### Pooled fecal sample used in the *in vitro* model

**Figure 1:**
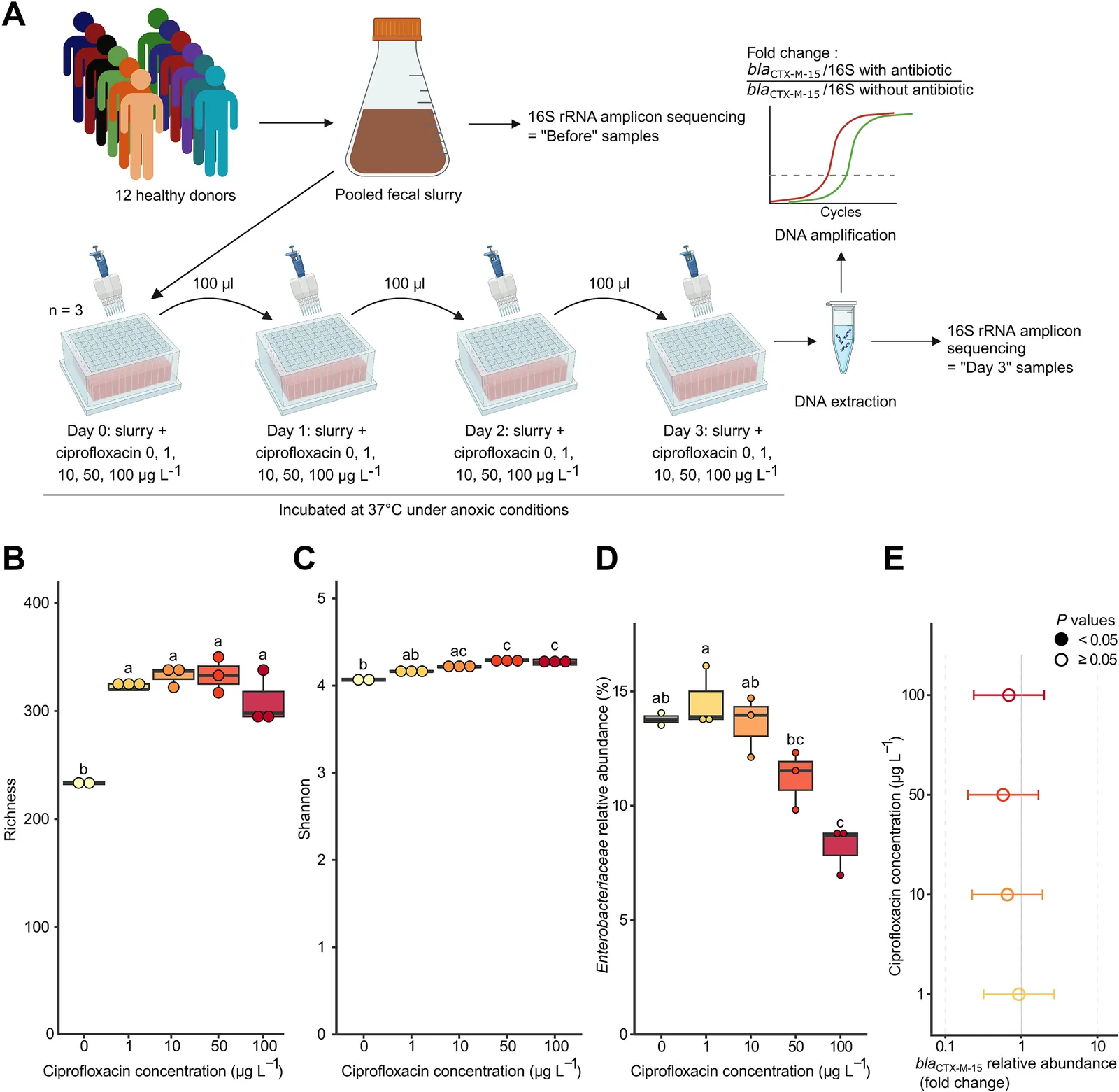
An *in vitro* model of the human gut microbiota. (A) a schematic overview of the experimental model. (B) Observed ASV richness and (C) evenness of the bacterial community, and (D) relative abundance of Enterobacterales after exposition to low concentrations of ciprofloxacin. (E) Relative abundance of *bla*_CTX-M-15_ after exposition to low concentrations of ciprofloxacin. Data points represent fold-changes of *bla*_CTX-M-15_ compared to the antibiotic-free control, normalized to the 16S rRNA gene (“16S”) copy number. The x-axis is in logarithmic scale. Empty circles indicate no significant differences relative to the control. Horizontal bars depict 5%–95% confidence intervals. *N* = 3 for all assays, except for Day-3 samples without ciprofloxacin where *N* = 2 after to removal of an outlier. For b, c, and d, statistical significance was evaluated using one-way ANOVA, followed by Tukey’s post-hoc test, and significant differences are indicated by distinct letters above boxplots. Source data and exact *P* values are provided in the source data file. Created with BioRender.com.

Feces samples were collected from 15 healthy volunteers (20–60 years old) who had not received antibiotic treatment in the previous 12 months (Fig. 1a). Volunteers provided a feces sample stored in a sterile container with an AnaeroGen Compact bag (ThermoFisher, San Jose, CA, USA) to create anoxic conditions, as recommended by the International Human Microbiome Standards (https://human-microbiome.org). Using culture on selective agar media, all feces samples were tested for the absence of the most frequent antibiotic-resistant pathogens (methicillin-resistant *Staphylococcus aureus*, vancomycin-resistant enterococci, carbapenemase-producing *Acinetobacter baumannii*, and *Pseudomonas aeruginosa*, and Enterobacterales producing carbapenemase or extended-spectrum β-lactamase). We discarded from the study three volunteers whose fecal sample were positive with at least one of these antibiotic-resistant pathogens. The remaining twelve fecal samples came from donors (7 females, 5 males) ranging in age from 21 to 52 years. Samples were diluted 1:5 w/v in water and gauze-filtered. Within 24 h after collection, we pooled equivalent volumes of each fecal slurry and stored the mixed feces at −80°C until used. Such conservation procedure efficiently preserves microbial ecosystem [24].

To ensure that the pooled fecal slurry did not contain concentrations of antibiotics that could interfere with the experiments, we quantified clinically relevant and persistent antibiotics (ciprofloxacin, norfloxacin, ofloxacin, erythromycin, trimethoprim, sulfamethoxazole, and chloramphenicol) using a method described in Supplementary data. The concentration of all tested antibiotics was below the detection limit (1 μg L^−1^) in the pooled fecal slurry.

All the experiments were conducted with the same batch of pooled fecal slurry. The study was approved by an ethics committee prior to study commencement (CPP Sud Ouest et Outre Mer IV, France, reference 2022-A02602–41).

### Collection of exogenous bacterial isolates

We selected a range of Gram-negative bacterial pathogens with an intestinal tropism to inoculate the pooled fecal slurry. A total of 28 isolates belonging to the most frequent multi-drug resistant clones spreading globally included strains of *Escherichia coli* (*n =* 10), *Klebsiella pneumoniae* (*n =* 6), *Enterobacter hormaechei* (*n =* 5), *A. baumannii* (*n =* 4), and *P. aeruginosa* (*n =* 3) (Table 1; Table S1) [3]. All strains were isolated from patients except *E. coli* ST131 CTX-M-15 isolated from urban wastewater.

**Table 1:** Characteristics of the bacterial strains used to inoculate the pooled fecal slurry in the present study. The origin, full resistance profile, and resistance determinants for each strain are available in Table S1.

**Species**	**Sequence type** ^**a**^	**Phylo-group**	***fimH* type**	**β-lactamase(s) with an** **extended spectrum (location)**^b^	**Ciprofloxacin MIC** ^c^ **(mg L**^**−1**^**)**
*E. coli*	ST131	B2	30	**CTX-M-15 (pl.)**	32
	ST10	A	215	**CTX-M-15 (chr.)**	128
	ST131	B2	30	**CTX-M-27 (chr.)**	32
	ST141	B2	5	**CTX-M-14 (chr.)**	0.008
	ST38	D	5	**CTX-M-14 (chr.)**, DHA-1 (chr.)	0.125
	ST88	C	303	**CTX-M-1 (chr.)**	16
	ST12	B2	5	**OXA-48 (pl.)**	0.008
	ST127	B2	2	**OXA-48 (pl.)**	0.008
	ST410	C	24	**NDM-1 (pl.)**, CTX-M-15 (pl.), CMY-2 (chr.)	128
	ST48	A	34	**IMP-1 (pl.)**	0.125
*K. pneumoniae*	ST307	–	–	**CTX-M-15 (pl.)**, SHV-28 (chr.)	64
	ST107	–	–	**CTX-M-9 (pl.)**, TEM-24 (pl.)	4
	ST45	–	–	**OXA-48 (pl.)**, CTX-M-14 (pl.)	0.015
	ST20	–	–	**NDM-1 (pl.)**, CTX-M-15 (pl.), SHV-187 (chr.)	2
	ST636	–	–	**VIM-1 (pl.)**	0.5
	ST11	–	–	**KPC-3 (pl.)**, CTX-M-15 (pl.), DHA-1 (pl.)	>128
*E. hormaechei*	ST114	–	–	**CTX-M-15 (pl.)**	64
	ST78	–	–	**CTX-M-14 (pl.)**	2
	ST171	–	–	**NDM-1 (pl.)**, CTX-M-15 (chr.)	64
	ST133	–	–	**OXA-48 (pl.)**	0.008
	ST171	–	–	**KPC-3 (pl.)**	32
*A. baumannii*	ST2	–	–	**OXA-23 (pl.)**	64
	ST578	–	–	**OXA-23 (pl.)**, OXA-58 (chr.)	64
	ST537	–	–	**OXA-24 (pl.)**	32
	ST821	–	–	**NDM-1 (pl.)**, OXA-420 (pl.)	64
*P. aeruginosa*	ST235	–	–	**VIM-1 (pl.)**	16
	ST244	–	–	**IMP-1 (pl.)**	32
	ST235	–	–	**IMP-19 (chr.)**	64

a Sequence type, see Table S1 for MLST schemes.

b *bla* genes quantified by qPCR are shown in bold. The replicons carrying the encoding genes are in brackets: pl., plasmid; chr., chromosome.

c MIC measured by agar dilution method following the CLSI recommendations [25].

### Antibiotic susceptibility profiles of exogenous bacterial isolates

MICs of the antibiotics were determined with the agar dilution method following the CLSI recommendations and using *E. coli* ATCC 25922 and *S. aureus* ATCC 25923 as controls [25]. We tested the following compounds with the concentration range in brackets: ciprofloxacin (0.002–128 mg L^−1^), enrofloxacin (0.002–128 mg L^−1^), norfloxacin (0.002–128 mg L^−1^), ofloxacin (0.002–128 mg L^−1^), erythromycin (0.125–256 mg L^−1^), tylosin (4–2048 mg L^−1^), tetracycline (0.125–256 mg L^−1^), oxytetracycline (0.125–256 mg L^−1^), chlortetracycline (0.125–256 mg L^−1^), doxycycline (0.125–256 mg L^−1^), trimethoprim (0.5–128 mg L^−1^), trimethoprim-sulfamethoxazole (expressed as TMP:SMX in the ratio 1:19; 0.25–256 mg L^−1^), sulfadiazine (4–2048 mg L^−1^), sulfadimidine (4–2048 mg L^−1^), sulfachlorpyridazine (4–2048 mg L^−1^), sulfadimethoxine (4–2048 mg L^−1^), chloramphenicol (2–256 mg L^−1^).

### Genotypic characterization of exogenous bacterial isolates

The genomes of all isolates were sequenced. Briefly, all isolates were isolated from an overnight culture on Mueller-Hinton agar (Bio-Rad) and extracted with a QIAmp DNA Minikit (Qiagen, Hilden, Germany) according to the manufacturer’s instructions. DNA was sequenced on a NextSeq System (Illumina, San Diego, CA, USA), generating 150-bp paired-end reads with a mean coverage of 80X. Sequencing data are available in the NCBI BioProjects PRJNA1101420 and PRJNA975705. MLST analyses were performed using pyMLST software [26]. *Escherichia coli* strains were also subjected to *fimH* typing and phylogrouping *in silico* with FimTyper [27] and with ClermonTyping [28], respectively. We identified genes coding for AMR mechanisms and resistance-associated point mutations using AMRfinderPlus [29]. Genes encoding ꞵ-lactamases with an extended spectrum were located using mlplasmids [30]. Resistance phenotypes, resistance genes, the replicon (chromosome or plasmid) carrying the *bla*_ESBL_ and/or *bla*_CARBA_, typing data, and NCBI accession numbers for all isolates are detailed in Table S1. The isolates tested carried one (*n =* 18), two (*n =* 7), or three (*n =* 3) *bla*_ESBL_ and/or *bla*_CARBA_. To assess strain proliferation, we quantified the *bla* genes encoding CTX-M-1, −9, −14, −15, −27, OXA-23, −24, −48, IMP-1, −19, VIM-1, NDM-1, and KPC-3 (Table 1).

### Antibiotics tested in the pooled fecal slurry

In a first series of experiments, eleven antibiotics (ciprofloxacin, norfloxacin, ofloxacin, danofloxacin, enrofloxacin, erythromycin, chloramphenicol, tetracycline, sulfamethoxazole, trimethoprim, and cotrimoxazole) were each tested separately at six concentrations (0.25, 0.5, 1, 10, 50, and 100 μg L^−1^). We selected sulfamethoxazole as a representative of the sulfonamides and tetracycline as a representative of the tetracycline class. Secondly, a mixture of 15 antibiotics used in veterinary medicine or that are preferred as veterinary antibiotics (sulfadiazine, sulfamethoxazole, sulfadimidine, sulfachlorpyridazine, trimethoprim, sulfadimethoxine, tetracycline, oxytetracycline, chlortetracycline, doxycycline, ciprofloxacin, enrofloxacin, ofloxacin, erythromycin, and tylosin) at concentrations of 10 or 20 μg L^−1^ each. These concentrations were based on those found in the feces of healthy people that were not undergoing antibiotic therapy [21–23]. All chemicals were purchased from Sigma-Aldrich (St. Louis MO, USA).

### *In vitro* model of gut microbiota

**Figure 2:**
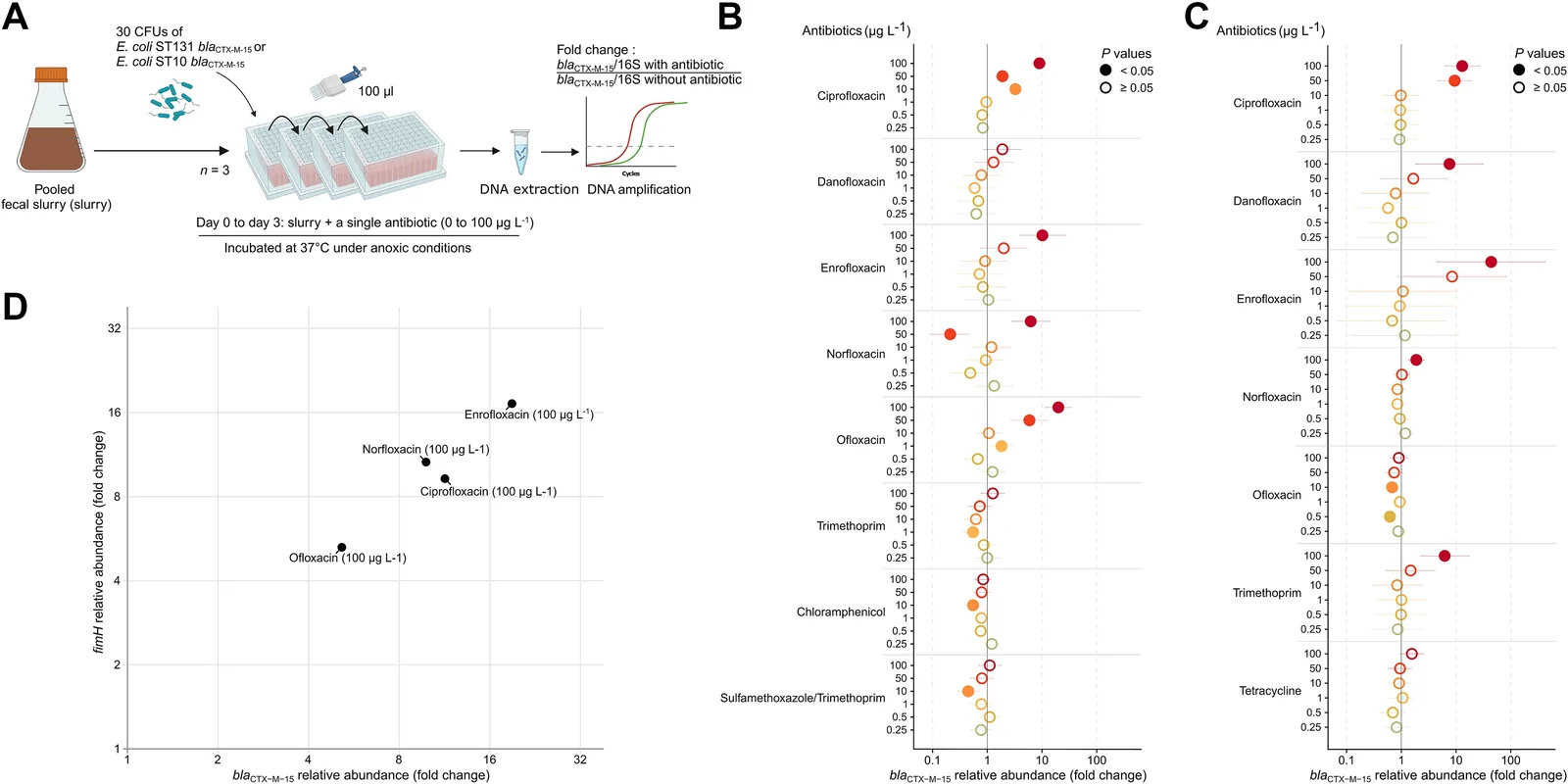
Low concentrations of fluoroquinolones or trimethoprim enrich a pooled fecal slurry with major epidemic strains of *E. coli* CTX-M-15. (A) A schematic overview of the experimental model. Relative abundance of *bla*_CTX-M-15_ provided by (B) *E. coli* ST131, or (C) *E. coli* ST10. The dots show fold-changes of *bla*_CTX-M-15_ relative abundance compared to the antibiotic-free control, normalized to the 16S rRNA gene (“16S”) copy number. The x-axis is in logarithmic scale. Empty circles indicate no significant differences relative to the control, and filled dots denote a *P* value <.05. Horizontal bars represent 5%–95% confidence intervals for each condition. *N* = 3 for all assays. Eleven antibiotics (ciprofloxacin, norfloxacin, ofloxacin, danofloxacin, enrofloxacin, erythromycin, chloramphenicol, tetracycline, sulfamethoxazole, trimethoprim, and cotrimoxazole) were tested on each strain but only antibiotics that significantly (likelihood ratio test; *P* < .05) affected the model were further tested with a Dunnett test (*P* < .05). (D) Correlation between *E. coli* ST131-specific *fimH* gene and *bla*_CTX-M-15_ under fluoroquinolone exposure (at 0 vs. 100 μg L^−1^). Fold changes of *fimH* (y-axis) and *bla*_CTX-M-15_ (x-axis) were quantified by qPCR, normalized to 16S rRNA gene copy number, and expressed relative to antibiotic-free controls for each drug. Points represent mean values for each condition; control samples (0) cluster at unity. The dashed diagonal indicates equal enrichment of both targets, and deviations reflect relative imbalance. Source data and exact *P* values in the source data file. Created with BioRender.com.

The potential impact of low concentrations of antibiotics on the establishment of antibiotic resistant Gram-negative pathogens into the gut microbiota following inoculation with exogenous bacteria was determined in an *in vitro* gut model (Fig. 1a). For each assay, 2 ml of the pooled fecal slurry supplemented with various antibiotic additions were inoculated with 30 colony-forming units (CFUs; Fig. 2a). To calibrate the inoculum, a bacterial suspension of 0.5 McFarland (corresponding to ~10^8^ CFU/mL) was prepared in saline solution from colonies grew at 37°C overnight on Luria-Bertani Agar (LBA), further diluted to 10^3^ CFU/ml. Each assay was inoculated with 50 μL of the suspension. As a control, 50 μL of each inoculum were plated in triplicate on LBA plates for CFU quantification. All inoculum were comprised between 27 and 37 CFUs.

The inoculated pooled fecal slurry was incubated in 96-well deep plates (Macherey-Nagel, Düren, Germany) under anoxic conditions (Whitley A35 Chamber, 85.0% N_2_, 10.0% H_2_, 5.0% CO_2_) at 37°C. Following 24 h of incubation, 100 μl of the slurry was transferred to 2 ml of fresh slurry supplemented with the same concentrations of antibiotics. The procedure was repeated a second time and following 72 h of incubation, 1-ml aliquots of the slurries were collected for DNA extraction. The purpose of this daily transfer was to replicate the ingestion of food containing antibiotics and to maintain antibiotic concentrations over the course of the tests. In all experiments, controls that received no antibiotics were included and all assays were done at least in triplicate. Two general experimental approaches were used. First, we evaluated the impact of 11 antibiotics at varying concentrations on pooled fecal slurry inoculated with either *E. coli* ST131 CTX-M-15 or *E. coli* ST10 CTX-M-15. Second, we tested the impact of ciprofloxacin at 10 or 50 μg L^−1^ on pooled fecal slurry inoculated with one of the 28 Gram-negative pathogens (Table 1). Third, we tested the impact of a mixture of 15 veterinary antibiotics at either 10 or 20 μg L^−1^ (each) in similar conditions.

### Analysis of the microbiota composition

Raw DNA extracts were submitted for 16S rRNA gene sequencing at Microsynth AG (Balgach, Switzerland). Briefly, the hypervariable regions V3-V4 of the 16S rRNA genes were amplified using the primers 341F (5’-CCTACGGGNGGCWGCAG-3′) and 805R (5’-GACTACHVGGGTATCTAATCC-3′) [31]. PCR amplicons were sequenced on a MiSeq System (Illumina). Quality control, read pair assembly, and chimera removing were performed using the DADA2 pipeline [32] on RStudio environment. Filtered sequences displaying 100% identity were gathered into ASV. A representative sequence from each ASV was then compared with reference sequences from the SILVA rRNA database project (SILVA database v. 138.1) [33]. The final product is a list of ASVs detected, their taxonomy, and their distribution (relative abundances) in each sample (Table S2). Raw sequencing reads have been deposited in the European Nucleotide Archive under the BioProject PRJEB83439.

### *bla*_ESBL_ and *bla*_CARBA_ gene quantification

To monitor the potential enrichment of resistant pathogens, we measured the relative concentration of the *bla*_ESBL_ or *bla*_CARBA_ genes their carried (Table 1, Table S1). Total DNA was extracted from the pooled fecal slurry with the QIAmp kit (Qiagen) according to the manufacturer’s protocol with two additional centrifugation steps (10 min, 8000 *g*) to pellet fecal material, including bacteria before lysis, and to discard fecal material after lysis. DNA solutions were diluted 10-fold prior to gene target quantification. In each experiment, a single *bla*_ESBL_ or *bla*_CARBA_ gene was quantified using specific Taqman qPCR assay normalized to the amount of 16S rRNA genes (ThermoFisher Scientific; Table 1, Table S1). We represented the results as fold change with a 95% confidence interval (CI_95%_).

### *E. coli* ST131 core gene quantification

We designed a TaqMan qPCR assay targeting a chromosomal *E. coli* ST131-specific marker to enable direct comparison with the abundance of the plasmid-borne *bla*_CTX-M-15_. The chromosomal locus was identified by comparative pangenome analysis of 200 *E. coli* genomes using PanExplorer [34], followed by verification of sequence specificity by BLAST. Its chromosomal localization in the studied *E. coli* ST131 strain was confirmed using PlasmidHunter [35]. Primers and a hydrolysis probe were designed using Primer3 [36]. The assay targets the *fimH* gene with oligonucleotides given in Table S1. Target gene abundances (*fimH* and *bla*_CTX-M-15_) were normalized to the 16S rRNA gene. For each antibiotic, normalized values were expressed relative to the corresponding untreated control. The correlation between *fimH* and *bla*_CTX-M-15_ relative concentrations was assessed by computing their fold-change ratio and visualized on log2-scaled plots.

### Statistical analysis

The effects of treatments on the microbiota composition were assessed within the R statistical computing environment. Alpha diversity, including richness, Simpson, and Shannon indices, was estimated with the phyloseq package (v1.42.0) [37]. To determine significant differences among samples, we performed ANOVA tests (equivalent to *t*-test in this context) comparing samples before and after the experiment without treatment (0 μg L^−1^ of antibiotics), and between samples on day 3 of the experiment under different antibiotic concentrations (0 to 100 μg L^−1^). The homogeneity of variance was assessed using the F-test and the Bartlett test, and the Shapiro–Wilk test was performed to verify the normality of the data distribution. Post-hoc comparisons between treatments were conducted using Tukey’s Honest Significant Difference method.

Differences in bacterial community composition between samples were visualized by principal coordinate analysis. ASV sequences were first aligned with the MUSCLE method [38] using the msa package [39]. DNA distances were then calculated using the Kimura 2-parameter model to perform a neighbor-joining tree estimation. This analysis was performed using the ape [40], phangorn [41], and phyloseq packages [37]. Based on the weighted UniFrac distance metric, compositional differences were visualized in principal coordinate analysis using the ggplot2 package [42]. Significance of community differences was tested by a PERMANOVA test using the adonis2 function from vegan package (v2.6–4) [43].

To test whether traces of antibiotics could enrich the gut microbiota with *bla*_ESBL/CARBA_ carriers, statistical test was performed on R (4.2.2) using lme4 [44], multcomp [45], and ggplot2 packages [42]. A linear mixed model was built to evaluate the impact of different antibiotics, using repetition as a random effect. A likelihood ratio test was used to determine the significance of antibiotics in the model. For antibiotics with a *P* value less than 0.1, a Dunnett test was performed to determine the effect of different antibiotic concentrations in comparison to the absence of antibiotics.

We evaluated the relationship between ciprofloxacin MICs, lowest observable effect concentrations (LOECs) of the antibiotic mix, with the MIC of each of the compounds and with the number of genetic determinants of resistance to these compounds using a non-parametric Wilcoxon test.

## Results

### Implementation of an *in vitro* model of the human gut microbiota

We developed an *in vitro* model of the human gut microbiota to test whether traces of antibiotics enrich the human gut microbiome with antibiotic-resistant pathogens (Fig. 1a). To test whether the richness and diversity of the microbial flora were preserved in the model, we compared the composition of the flora before and after 72 h of incubation under anoxic conditions, using 16S rRNA gene sequencing. Multivariate analysis revealed that α-diversity decreased between Day 0 and Day 3 (Fig. S1). Whereas incubation of the fecal slurry without antibiotic shifted the community composition, with an enrichment of Enterobacterales [24] (Fig. S2, Fig. S3), it maintained an overall high α-diversity (Fig. S1 and Fig. S4)).

Incubation of the pooled fecal slurry with traces of ciprofloxacin could alter the richness and eveness of the flora, reducing its resistance to colonization, potentially favoring the implantation of antibiotic-resistant pathogens. Likewise, *bla* genes undetected by culture methods could be enriched. To test this, we exposed for 72 h the fecal slurry to ciprofloxacin at concentrations of 1, 10, 50, and 100 μg L^−1^ and further assessed its richness, evenness, and the *bla*_CTX-M-15_ concentration (the most common *bla*_ESBL_ in Gram-negative bacilli). We found that incubation with ciprofloxacin increased the richness and evenness of the gut microbiota (Fig. 1b and c) without promoting the growth of Enterobacterales (Fig. 1d). The absence of *bla*_CTX-M-15_ enrichment in the fecal slurry alone (Fig. 1e) showed that *bla*_CTX-M-15_ measured in the following experiments originated from inoculated exogenous resistant pathogens.

### Low concentrations of antibiotics promote the implantation of epidemic clones of *E. coli* producing CTX-M-15 in the gut microbiota

We determined the LOECs of eleven different antibiotics (chloramphenicol, ciprofloxacin, danofloxacin, enrofloxacin, norfloxacin, ofloxacin, erythromycin, tetracycline, trimethoprim, sulfamethoxazole, and cotrimoxazole) used in human or veterinary medicine on the relative abundance of two major clones of *E. coli* producing CTX-M-15 into the fecal slurry (Fig. 2a). Within a drug concentration range of 0.25 to 100 μg L^−1^, the LOECs of ciprofloxacin, enrofloxacin, norfloxacin, ofloxacin for *E. coli* ST131 CTX-M-15 were 10, 100, 100, and 1 μg L^−1^, respectively (Fig. 2b). Hence, compared to the antibiotic-free control the relative concentration of the pathogen increased 3.27 fold (CI_95%_ 2.50–4.28) with 10 μg L^−1^ of ciprofloxacin, 10.17 fold (3.85–26.86) with 100 μg L^−1^ of enrofloxacin, 6.22 fold (2.82–13.73) with 100 μg L^−1^ of norfloxacin, and 1.82 fold (1.06–3.11) with 1 μg L^−1^ of ofloxacin (Fig. 2b). Similarly, the LOECs of ciprofloxacin, enrofloxacin, norfloxacin, danofloxacin, and trimethoprim on the relative abundance of *E. coli* ST10 CTX-M-15 were 50, 100, 100, 100, and 100 μg L^−1^, respectively (Fig. 2c). Following inoculation with *E. coli* ST10 CTX-M-15, its relative abundance in the model increased 9.44-fold (4.61–19.34) with 50 μg L^−1^ of ciprofloxacin, 43.85-fold (4.53–424.68) with 100 μg L^−1^ of enrofloxacin, 1.88-fold (1.37–2.60) with 100 μg L^−1^ of norfloxacin, 7.59-fold (1.85–31.13) with 100 μg L^−1^ of danofloxacin, and 6.20-fold (2.23–17.19) with 100 μg L^−1^ of trimethoprim (Fig. 2c). The LOECs of the other tested compounds could not be determined because they did not significantly influence the model (likelihood ratio test, *P* < .05, see Source Data).

The suitability of *bla* genes as markers of strain expansion was assessed by correlating their relative concentration with that of a chromosomal core gene. To maximize the chance of detecting a signal, we focused on conditions in which plasmid-borne *bla* genes were enriched >5-fold (i.e. with the pandemic strain *E. coli* ST131 producing CTX-M-15). We found that the ratio of *bla*_CTX-M-15_ to the *fimH* core gene abundance did not differ from 1 (one sample t-test, *P* = .52) (Fig. 2d). This suggests that horizontal transfer of *bla* genes was very limited or absent, and that *bla* genes were, in our experiments, appropriate markers of strain proliferation.

### Low concentrations of ciprofloxacin promote implantation of ESBL- and carbapenemase-producing Gram-negative pathogens in fecal slurry

**Figure 3:**
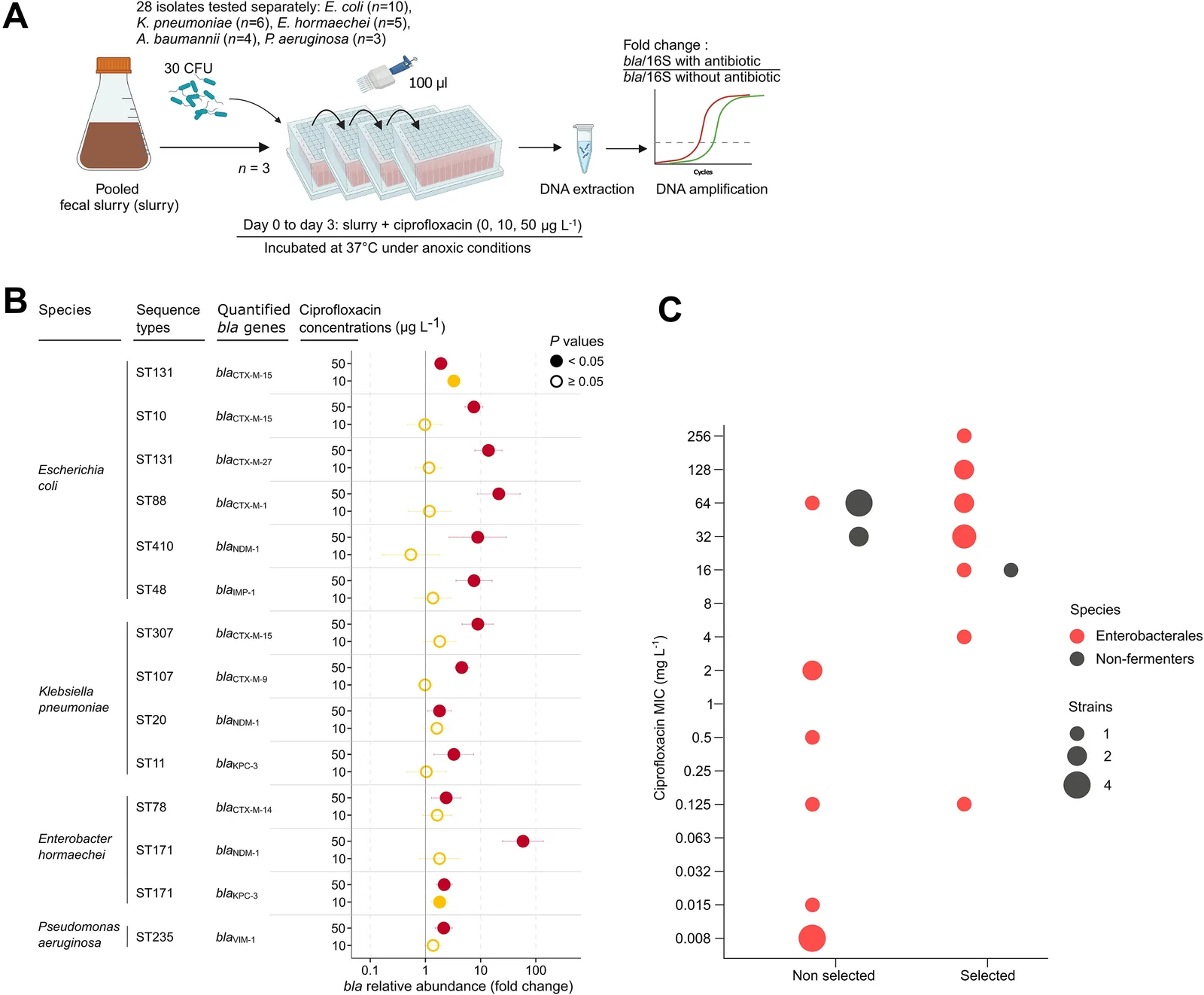
Low concentrations of ciprofloxacin enrich a pooled fecal slurry with pathogenic Gram-negative bacilli producing extended-spectrum β-lactamases and/or carbapenemases. (A) A schematic overview of the experimental model. (B) Relative abundance of *bla*_ESBL_ or *bla*_CARBA_. The dots show fold-changes compared to the antibiotic-free control, normalized to the 16S rRNA gene (“16S”) copy number. The x-axis is in logarithmic scale. Empty circles indicate no significant differences relative to the control, and filled dots denote a *P* value <.05. Horizontal bars represent 5%–95% confidence intervals for each condition. *N* = 3 for all assays. Antibiotics that significantly (likelihood ratio test; *P* < .05) affected the model were further tested with a Dunnett test (*P* < .05). Source data and exact *P* values are in the source data file. (C) Distribution of the strains depending on their enrichment by ≤50 μg L^−1^ of ciprofloxacin, their susceptibility to this antibiotic (measured by the MIC), and their taxonomic group. We tested the association between enrichment of *bla* and ciprofloxacin MICs with a non-parametric Wilcoxon test. Created with BioRender.com.

Because ciprofloxacin is widely used worldwide and had consistent low LOECs towards major epidemic clones of *E. coli* (Fig. 2b and c), we further tested the effect of dietary concentrations found in the colon of healthy humans (i.e. 10 and 50 μg L^−1^) [21, 22] in fecal slurry inoculated with each of the 26 other epidemic Gram-negative pathogens (Fig. 3a, Table 1). Ten μg L^−1^ of ciprofloxacin enhanced the abundance two *bla*_ESBL/CARBA_ Gram-negative pathogens out of 28 tested: *E. coli* ST131 CTX-M-15 (3.27-fold, 2.50–4.28) and *E. hormaechei* ST171 KPC-3 (1.81-fold, 1.30–2.52) (Fig. 3b). Fourteen out of the 28 Gram-negative pathogens carrying *bla*_ESBL/CARBA_ tested were enriched in the presence of 50 μg L^−1^ ciprofloxacin: six strains of *E. coli* (of ST131, ST10, ST88, ST410, and ST48), four strains of *K. pneumoniae* (ST307, ST107, ST20, ST11), three strains of *E. hormaechei* (one ST78 and two ST171), and one strain of *P. aeruginosa* ST235 (Fig. 3b). For instance, 50 μg L^−1^ of ciprofloxacin increased 13.87-fold (7.86–24.49) the relative abundance of *E. coli* ST131 *bla*_CTX-M-27_, 21.32-fold (8.75–51.95) that of *E. coli* ST88 *bla*_CTX-M-1_, and 58.61-fold (25.12–136.77) that of *E. hormaechei* ST171 *bla*_NDM-1_.

Overall, ciprofloxacin had a LOEC ≤50 μg L^−1^ for 14 of the 28 resistant pathogens tested. Furthermore, 13 of the 21 multidrug-resistant Enterobacterales tested (61.9%) and one of the seven (14.2%) non-fermenting Gram-negative bacilli (*P. aeruginosa* or *A. baumannii*) were enriched by 10 to 50 μg L^−1^ of ciprofloxacin. To identify the characteristics of the spiked antibiotic-resistant pathogens that might explain their enrichment under ciprofloxacin pressure in the fecal slurry, we assessed whether enrichment correlated with the ciprofloxacin MICs of the 28 strains and with the pathogen group (i.e. Enterobacterales or non-fermenting bacilli). We found that enrichment of the Enterobacterales in the fecal slurry was correlated with ciprofloxacin MICs, in contrast to non-fermenting bacilli, for which enrichment was independent from ciprofloxacin MICs (Fig. 3c). Ciprofloxacin exposure (from 1 to 100 μg L^−1^) increased the richness and evenness of the gut microbiota (Fig. 1b and c) but did not favor the Enterobacterales order (Fig. 1d). Overall, the enrichment of Gram-negative bacilli carrying *bla*_ESBL/CARBA_ in the *in vitro* model of gut microbiota by low concentrations of ciprofloxacin may rely on strain co-selection by ciprofloxacin depending on the species, rather than the disruption of the flora that opens up a niche for colonization.

### Veterinary antibiotics in combination promote implantation of multidrug-resistant Gram-negative pathogens carrying *bla*_ESBL/CARBA_ in fecal slurry

**Figure 4:**
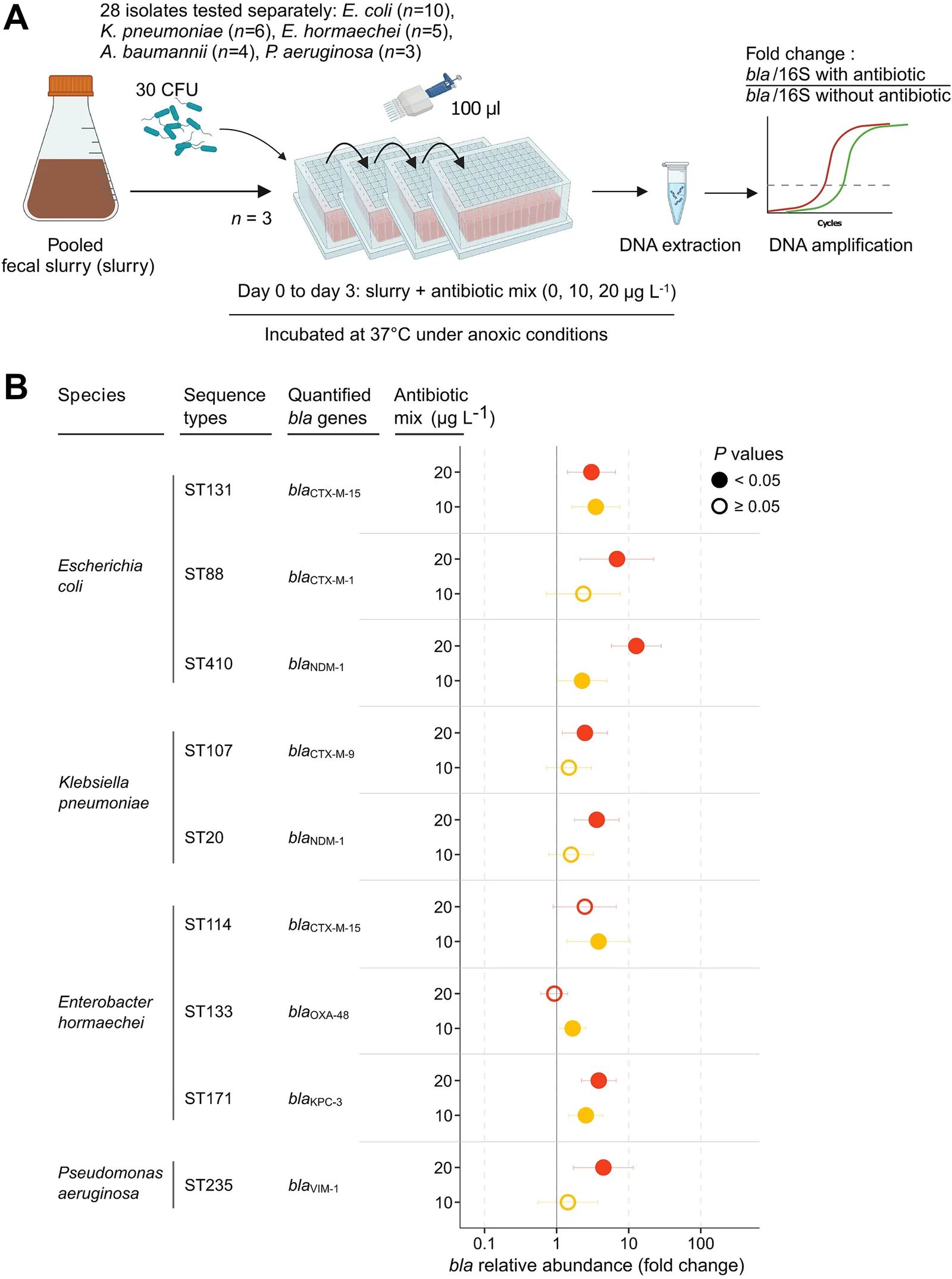
A mix of fifteen veterinary antibiotics enriches a pooled fecal with pathogenic Gram-negative bacilli producing extended-spectrum β-lactamases and/or carbapenemases. (A) A schematic overview of the experimental model. The mix was made of 15 antibiotics used in veterinary and/or human medicine (sulfadiazine, sulfamethoxazole, sulfadimidine, sulfachlorpyridazine, trimethoprim, sulfadimethoxine, tetracycline, oxytetracycline, chlortetracycline, doxycycline, ciprofloxacin, enrofloxacin, ofloxacin, erythromycin, and tylosin). (B) Relative abundance of *bla*_ESBL_ or *bla*_CARBA_. The dots represent fold-changes relative abundance compared to the antibiotic-free control, normalized to the 16S rRNA gene (“16S”) copy number. The x-axis is in logarithmic scale. Empty circles indicate no significant differences relative to the control, and filled dots denote a *P* value <.05. Horizontal bars represent 5%–95% confidence intervals for each condition. *N* = 3 for all assays. Only bacterial strains significantly (likelihood ratio test, *P* < .05) affected by the model were further tested with a Dunnett test (*P* < .05). Source data and exact *P* values are in the source data file. Created with BioRender.com.

The feces of healthy people can contain a mixture of antibiotics at low concentrations (~10–20 μg L^−1^) presumably originating from antibiotic-containing food [21–23]. We then tested the effect of a mixture of antibiotics used in veterinary medicine at 10 and 20 μg L^−1^ on the implantation in fecal slurry of 28 multidrug resistant Gram-negative pathogens carrying *bla*_ESBL/CARBA_ (Fig. 4a, Table 1). The mix of 15 antibiotics at 10 μg L^−1^ increased the relative abundance of five of the 28 (17.9%) pathogens carrying *bla*_ESBL/CARBA_. Namely, *E. coli* ST131 CTX-M-15 (3.49-fold, 1.63–7.51), *E. coli* ST410 NDM-1 (2.25-fold, 1.02–5.00), *E. hormaechei* ST114 CTX-M-15 (3.82-fold, 1.39–10.46), *E. hormaechei* ST133 OXA-48 (1.67-fold, 1.09–2.56), and *E. hormaechei* ST171 KPC-3 (2.55-fold, 1.47–4.42) (Fig. 4b). The mix with 20 μg L^−1^ of each antibiotic increased the relative abundance of seven of the 28 (25.0%) *bla*_ESBL/CARBA_ carrying pathogens tested. Namely, *E. coli* ST131 CTX-M-15 (3.04-fold, 1.41–6.53), *E. coli* ST88 CTX-M-1 (6.88-fold, 2.12–22.32), *E. coli* ST410 NDM-1 (12.75-fold, 5.76–28.26), *K. pneumoniae* ST107 CTX-M-9 (2.47-fold, 1.20–5.06), *K. pneumoniae* ST20 NDM-1 (3.60-fold, 1.77–7.35), *E. hormaechei* ST171 KPC-3 (3.84-fold, 2.21–6.67), and *P. aeruginosa* ST235 VIM-1 (4.46-fold, 1.72–11.55) (Fig. 4b). Altogether, 9 of the 28 (32.1%) resistant pathogens tested were enriched by ≤20 μg L^−1^ of the antibiotic mix.

Because the 28 strains tested had a wide range of resistance to the antibiotics of the mix (Table S1), we hypothesized that the degree of resistance of the strains to the antibiotics tested could account for the difference of behavior in the fecal slurry. For each of the 28 strains, MICs of all the 15 antibiotics present in the mix were measured and the genetic determinants of the resistance to these compounds were identified from sequencing data (Table S1). We tested the relationship between the LOECs of the antibiotic mix (10 or 20 μg L^−1^) with the MIC of each of the compounds and with the number of genetic determinants of resistance to these compounds, and found no correlation (non-parametric Wilcoxon test, *P* > .05).

## Discussion

In the present study, we evaluated the effects of low concentrations of antibiotics on the dynamics of *bla*_ESBL/CARBA_ carrying multidrug-resistant Gram-negative pathogens following their inoculation into an *in vitro* model of the human gut microbiota. We found that low antibiotic concentrations, comparable to those measured in the feces of humans exposed to antibiotics through dietary intake, increased the relative abundance of multidrug-resistant Gram-negative bacteria carrying *bla*_ESBL/CARBA_.

Ciprofloxacin concentrations of 10–50 μg L^−1^ increased the relative abundance of 14 of 28 resistant Gram-negative pathogens carrying *bla*_ESBL/CARBA_ inoculated into fecal slurry (Fig. 2a and b and Fig. 3b). These observations are in agreement with data indicating that exposure of complex environmental matrices to antibiotics far below clinical concentrations can increase the relative abundance of antibiotic resistant bacteria and the genes that they carry. For example, the relative abundance of *intI1*, a marker of antibiotic resistance, increased in a sewage microbial community supplemented with 11–16 μg L^−1^ of ciprofloxacin [46]. In addition, a resistant *E. coli* was enriched in a biofilm from wastewater supplemented with 2.5–10 μg L^−1^ of ciprofloxacin [47, 48]. In the present study, 100 μg L^−1^ of trimethoprim increased the relative abundance of *E. coli* ST10 CTX-M-15 (Fig. 2c), consistent with the LOEC of 63 μg L^−1^ for *intI1* measured in a sewage microcosm [46]. Despite the differences in microbial community composition and oxygen concentration between sewage and feces [49], the LOECs measured in these communities are in the same range. These results are in agreement with pure culture studies revealing that, in some instances, antibiotic concentrations hundreds of times lower than the MICs can select for and maintain antibiotic resistance [50].

The indigenous microflora of the colon provides an important host defense by inhibiting colonization with exogenous pathogenic microorganisms [5, 6]. Among the antibiotics tested that enrich exogenous resistant pathogens, fluoroquinolones were the most represented (Fig. 2b and c). We therefore examined shifts in bacterial composition of the fecal slurry incubated with low concentrations of a representant of this family, ciprofloxacin. Incubation with ciprofloxacin (1–100 μg L^−1^) increased the richness and evenness of the fecal slurry (Fig. 1b and c), presumably maintaining its resistance to colonization. This suggests that the reduction of dominant populations allowed previously rare taxa to expand. The enrichment of the gut microbiota with antibiotic-resistant bacteria requires antibiotic selection pressure and overcoming the colonization resistance conferred by the indigenous microbiota [9]. Indeed, the colonization resistance of the resident microbiota incubated with traces of fluoroquinolones appears to be maintained against ciprofloxacin-resistant non-fermenting bacilli belonging to species normally absent from the flora of healthy individuals (i.e. *P. aeruginosa* and *A. baumannii*) (Fig. 3b and c). In contrast, ESBL- or carbapenamase-producing Enterobacterales (belonging to commensal species) were enriched under low ciprofloxacin pressure, depending on their level of resistance to this compound (Fig. 3b and c). However, despite its high-level resistance to ciprofloxacin (MIC, 64 mg/L), *E. hormaechei* ST114 *bla*_CTX-M-15_ was not enriched (Fig. 3c, Source data). Such a discrepancy may rely on differences in nutrient utilization required for gut colonization [51]. In addition, low concentrations of trimethoprim favored the trimethoprim-resistant *E. coli* ST10 but not trimethoprim-susceptible *E. coli* ST131 (Fig. 2b and c; Table S1). Overall, our data suggest that the gut flora incubated with traces of fluoroquinolones (*i*) resist to the colonization of non-fermenters carrying *bla*_ESBL/CARBA_, and (*ii*) allow the enrichment of Enterobacterales carrying *bla*_ESBL/CARBA_ through a mechanism of strain co-selection.

Antibiotics are widely used in terrestrial and aquatic food-animal production systems for the prevention and treatment of bacterial infections, or in some jurisdictions for growth promotion [13]. Globally, terrestrial farm animal production uses about 100 000 tonnes of antibiotics annually [52]. This results in the contamination of food of animal origin with antibiotics [53]. A critically important farming practice is fertilization of crop ground with animal or human fecal waste, recycling valued nutrients and reducing the need for chemical fertilizers [14]. This practice entrains residues of antibiotics into these systems, potentially contaminating the harvested crops [11, 54, 55]. Additionally, some antibiotics are applied directly onto crops as pesticides for control of bacterial diseases [56, 57]. Irrigating crops with effluent from wastewater treatment plant is being widely adopted in areas that are becoming increasingly arid due to climate change, threatening food security [58–60]. The irrigated wastewater can contain antibiotic residues that are entrained into soil or directly onto the crop depending on the irrigation method [61, 62]. Overall, various widely employed farming practices have the potential to directly or indirectly contaminate plant- and animal-based foods with antibiotics.

Human exposure to food-borne antibiotics is regulated through mandated standards in jurisdictions where the governance structure is sufficiently robust to enforce them. For example, the European Union, the Codex Alimentarius Commission, and the Canadian Food Inspection Agency specify the maximum permissible concentration of an antibiotic in foodstuffs (maximum residue limit) and the maximum amount of antibiotic consumed (acceptable daily intake, ADI) [63–65]. These standards were developed considering impacts on various microbiological and human health endpoints. However, measured LOECs suggest that low concentrations of antibiotics can favor a resistant bacterium over its isogenic counterpart, calling into question the safety of the ADI [46]. Consistent with this, recent studies have shown that exposure of *Galleria mellonella* and murine models to one-tenth of the ADI induces fluoroquinolone resistance in gut-borne *K. pneumoniae* [66, 67]. In addition, *E. coli* strains with reduced susceptibility to ciprofloxacin are selected in the feces of healthy individuals exposed to this compound at the ADI [68]. Here, we found that the LOECs of ciprofloxacin measured in fecal slurry (10–50 μg L^−1^) was in the range of the gut concentration modeled after the ingestion of the ADI (~ 2–10 μg L^−1^) [69]. Furthermore, in a cohort of Chinese citizens that were not being treated with any antibiotics, fluoroquinolones were detected in the feces of 13.6% (19/140) of the individuals, with 22.3 μg kg^−1^ and 5099.8 μg kg^–1^ as median and maximum concentrations, respectively [21]. Likewise, the feces of 3%–21% of Chinese people who had not received antibiotic treatment contained multiple veterinary antibiotics (i.e. sulfonamides, tetracyclines, fluoroquinolones, and macrolides) most presumably carried by food [22, 23], with the total concentrations of veterinary antibiotics being 27 ± 33 μg kg^−1^ [23]. In the present study, the mix of these antibiotics at 10 and 20 μg L^−1^ could enrich for 23.8% and 28.6% of the resistant Enterobacterales tested, respectively (Fig. 4b).

The present study suggests that antibiotics consumed in food can favor the implantation in the gut of drug-resistant Gram-negative bacilli acquired from different sources (i.e. human, environment, animals). Our finding could reconcile the two a priori contradictory observations that a balanced digestive microbiota protects against the establishment of AMR [5, 6, 70] and the high prevalence of AMR carriage in human populations not treated with antibiotics [71]. At a country level, antibiotic consumption in animals is positively correlated with AMR in critical and high-priority human pathogens, as classified by the WHO [2]. Worryingly, antibiotic use in food-animal production is predicted to increase, with several geographic hotspots of antimicrobial use in Asia [52], where AMR carriage is already high in humans [2, 71, 72].

A large array of antibiotic compounds has been tested against two strains belonging to the major epidemic clones of *E. coli* producing CTX-M-15 (i.e. belonging to ST131 and ST10) [73]. The concentrations of antibiotics in our experiments were those predicted or measured in the feces of humans consuming antibiotic-containing food [21, 22, 69]. The *in vitro* model mimicked a contamination with a low inoculum and a daily intake of traces of antibiotics from food (Fig. 2a, Fig. 3a, Fig. 4a). We quantified *bla*_ESBL/CARBA_ instead of the resistant pathogens to take into account potential horizontal gene transfer of the resistance determinants. Our experimental model also responded to the limitations of murine experiments including the lack of reproducibility, recontamination due to coprophagy, ethical and animal welfare concerns, differences in the gut microbial composition relative to humans, and to the urgent need for predictive models of community dynamics under antibiotic pressure [9, 74]. However, it does not take into account the microbiota composition variation between individuals and host-related factors such as immune responses, diet, environmental exposure to other chemicals, treatment with other pharmaceuticals that can influence the implantation of AMR in humans [9]. The use of a mixture of feces from healthy donors circumvents the inter-individual variability complicating the interpretation of gut microbiota studies [75]. Overall, our *in vitro* model is relatively inexpensive, easy to use, allows replication of assays and limits the need for further testing in expensive animal models [74].

Humans are exposed to low doses of antibiotics through the consumption of contaminated water or animal- or plant-based foods containing antibiotic residues [10, 11]. The present study suggests that dietary consumption of some antibiotics can result in concentrations in the human colon sufficiently high to favor the implantation of exogenous antibiotic-resistant pathogens. On this basis, it would be prudent to revisit the permissible antibiotic concentrations in food, and critically evaluate agricultural practices that contaminate animal- and plant-based foods with antibiotics.

## Supplementary Material

Martak_et_al_Supplementary_Data_wrag008

Martak_et_al_Supplementary_Table_1_wrag008

Martak_et_al_Supplementary_Table_2_wrag008

Martak_et_al_Source_data_wrag008

Martak_et_al_Supplementary_Methods_wrag008

## Data Availability

Data available in Supplementary material. Raw sequencing reads have been deposited in the European Nucleotide Archive under the BioProject PRJEB83439.
