# Supplementary material for: Foodborne antibiotics enrich human gut microbiota with pathogens producing extended-spectrum β-lactamases and carbapenemases
Source: ISME J. 2026 Jan 26;20(1):wrag008. doi: 10.1093/ismejo/wrag008 (PMC12915577; doi:10.1093/ismejo/wrag008)
Supplement: Martak_et_al_Supplementary_Data_wrag008 [file martak_et_al_supplementary_data_wrag008.docx]

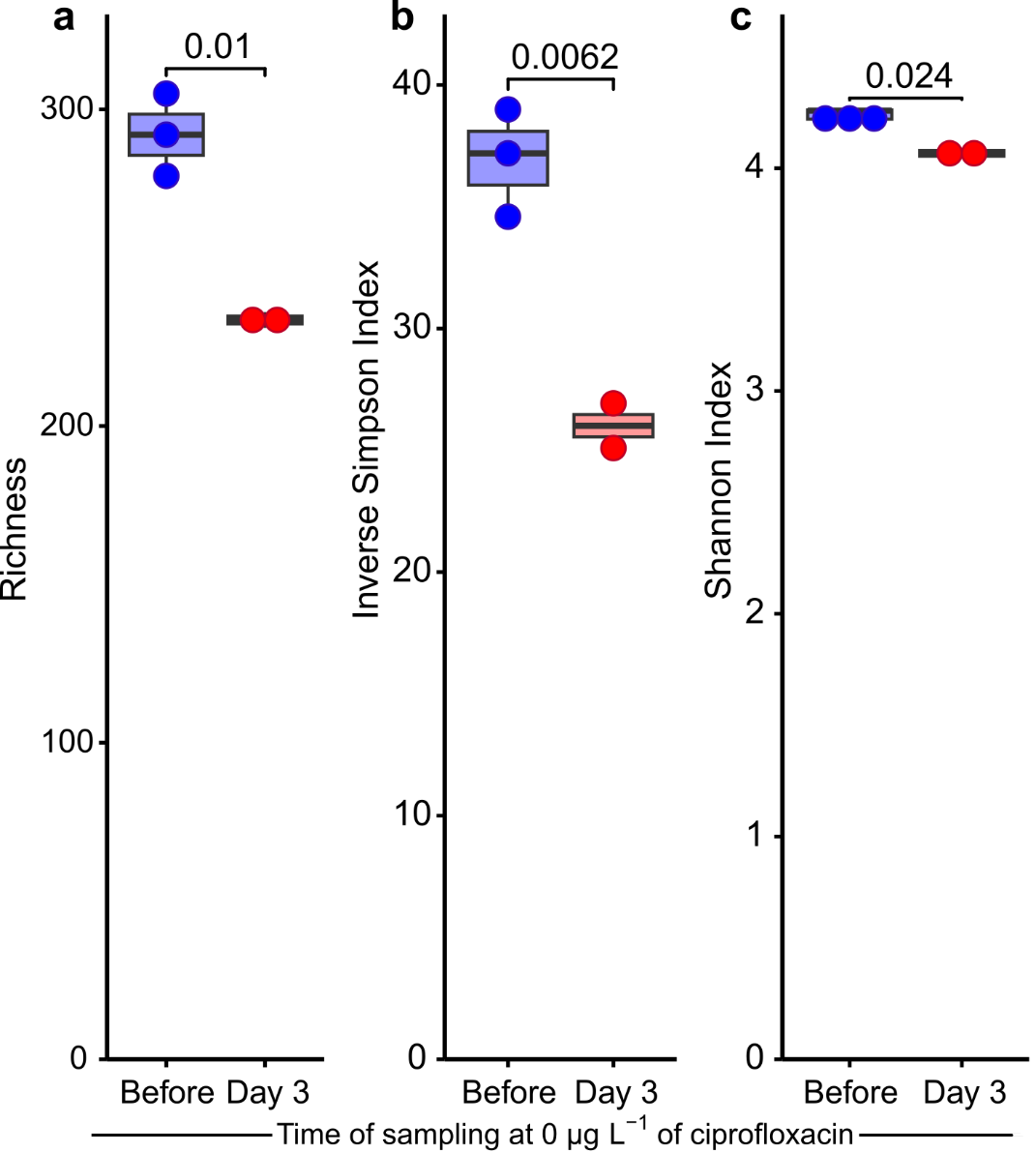


**Supplementary Fig. 1. Comparison of alpha diversity between gut microbiota samples collected before and during the experiment without antibiotic.** Boxplots represent different diversity indices: the left panel represents the observed ASV richness (taxonomic abundance), the middle panel displays the inverse Simpson index, and the right panel shows Shannon index. Blue boxplots correspond to samples taken from the raw frozen stool, while red boxplots represent samples collected after three days of incubation without antibiotic. We assessed statistical significance using t-tests with *P* values indicated above the boxplots.


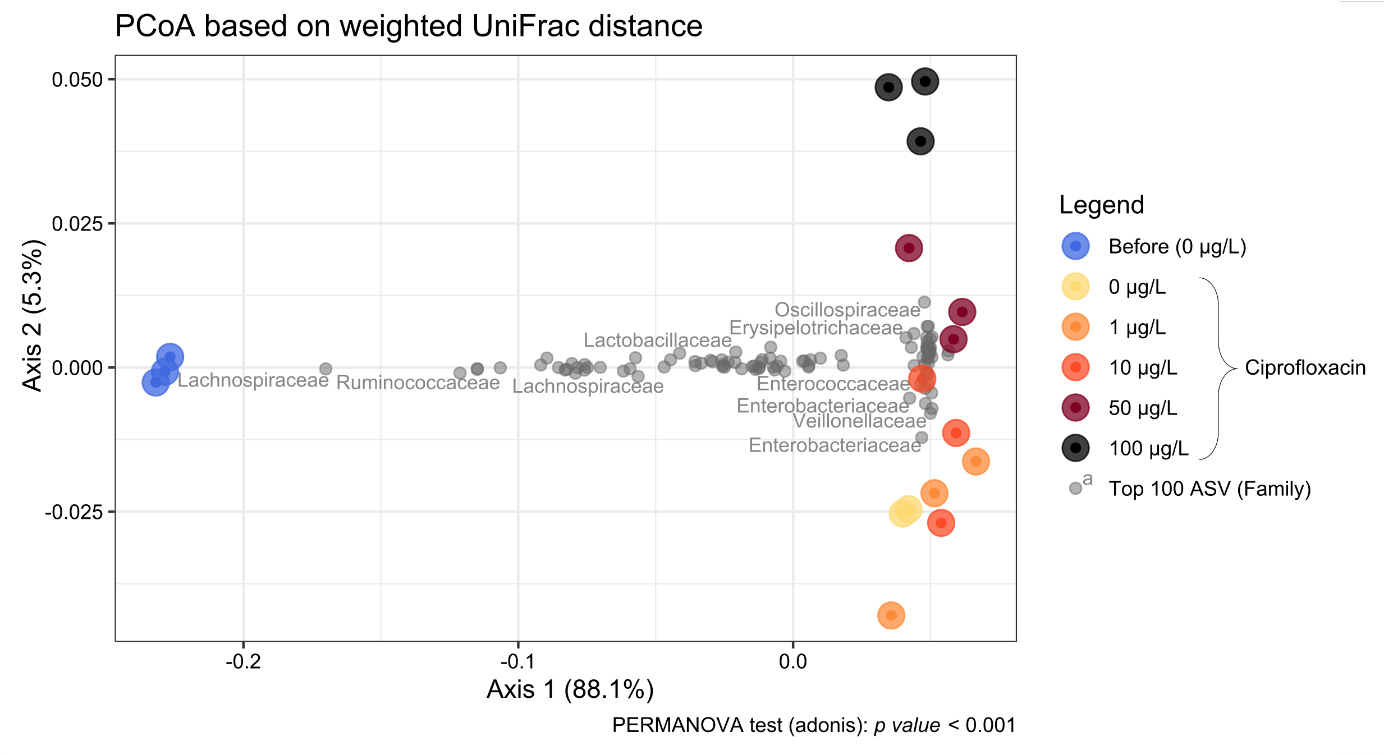


PERMANOVA test (adonis) : *P* value < 0.001

**Supplementary Fig. 2. Microbial composition of the fecal slurry changes over time and after exposure to low concentrations of ciprofloxacin.** Principal coordinate analysis is based on the weighted UniFrac distance calculated using ASV relative abundances, derived from a neighbor-joining tree estimation. **Samples** are represented as colored circles: blue represents fecal bacterial communities collected from the raw frozen stool, while shades from yellow to black represent increasing antibiotic concentrations. The green circles show the top 100 most abundant ASVs, with family names labeled where space permits. The variance explained by each axis is indicated as a percentage in parentheses. A PERMANOVA test performed using the *adonis2* function in R revealed significant differences in microbial composition across the conditions tested.


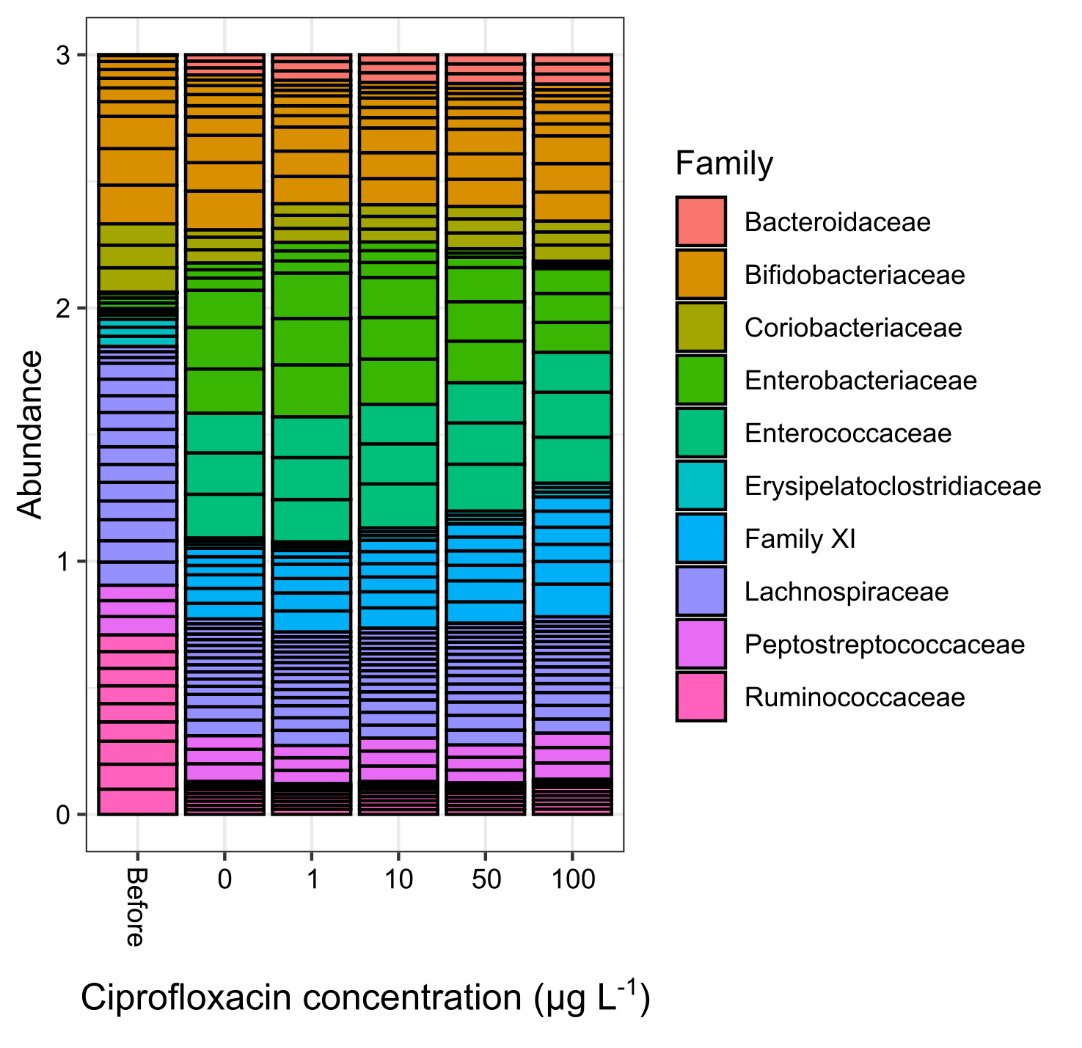


**Supplementary Figure 3. Average relative abundance of the bacterial family of the fecal slurry samples before the experiment and** **after exposure to low concentrations of ciprofloxacin**. Each relative abundance is derived from the average ASV abundance of three replicates.


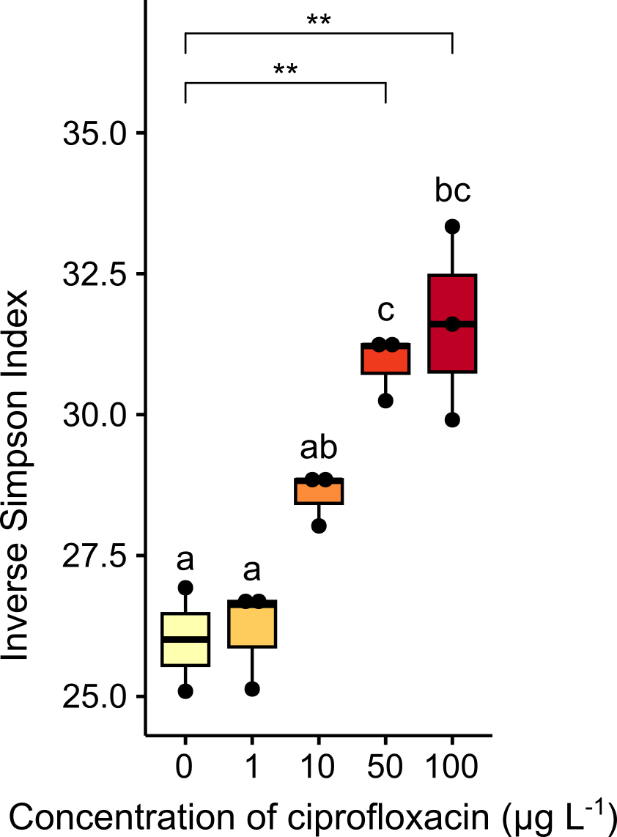


**Supplementary Figure 4: Comparison of alpha diversity between gut microbiota samples exposed at different concentrations of ciprofloxacin.** The boxplots show the inverse Simpson index. Statistical significance was assessed using one-way ANOVA followed by Tukey's post-hoc test to compare treatment groups. Differences between treatment groups are symbolized by distinct letters (a, b, c) above each boxplot; groups sharing a letter are not significantly different. Comparisons between the control group (0 µg L^-1^, no antibiotic) and other treatments are represented by *P* value significance levels: * (*P* < 0.05), ** (*P* < 0.01), *** (*P* < 0.001).

**Supplementary Methods**

Ciprofloxacin, norfloxacin, ofloxacin, erythromycin, trimethoprim, sulfamethoxazole, and chloramphenicol were quantified in the pooled fecal slurry using a Vanquish UHPLC system coupled to a Q-Orbitrap-HRMS mass spectrometer equipped with a heated electrospray ionization probe (HESI II) (Thermo FisherScientific). Chromatographic separation was performed on an ACQUITY UPLC BEH C18 column (50 × 2.1 mm, 1.7 µm) from Waters (Milford, MA, USA). The mobile phase consisting of (A) 0.1% formic acid in water and (B) acetonitrile with 0.1% formic acid was delivered at the flow rate of 0.5 mL min^−1^. Extraction was performed with methanol and a 4-µL aliquot of the extract was injected. Full scan data were acquired at a mass resolving power of 60,000 FWHM with positive ionization mode and the *m/z* scan range was 100–1000. The limit of quantification was 1 µg L^-1^ for each antibiotic.
