# Supplementary material for: Foodborne antibiotics enrich human gut microbiota with pathogens producing extended-spectrum β-lactamases and carbapenemases
Source: ISME J. 2026 Jan 26;20(1):wrag008. doi: 10.1093/ismejo/wrag008 (PMC12915577; doi:10.1093/ismejo/wrag008)
Supplement: Martak_et_al_Supplementary_Methods_wrag008 [file martak_et_al_supplementary_methods_wrag008.docx]

**Supplementary Methods**

Ciprofloxacin, norfloxacin, ofloxacin, erythromycin, trimethoprim, sulfamethoxazole, and chloramphenicol were quantified in the pooled fecal slurry using a Vanquish UHPLC system coupled to a Q-Orbitrap-HRMS mass spectrometer equipped with a heated electrospray ionization probe (HESI II) (Thermo FisherScientific). Chromatographic separation was performed on an ACQUITY UPLC BEH C18 column (50 × 2.1 mm, 1.7 µm) from Waters (Milford, MA, USA). The mobile phase consisting of (A) 0.1% formic acid in water and (B) acetonitrile with 0.1% formic acid was delivered at the flow rate of 0.5 mL min^−1^. Extraction was performed with methanol and a 4-µL aliquot of the extract was injected. Full scan data were acquired at a mass resolving power of 60,000 FWHM with positive ionization mode and the *m/z* scan range was 100–1000. The limit of quantification was 1 µg L^-1^ for each antibiotic.
